# Intermittent control models of human standing: similarities and differences

**DOI:** 10.1007/s00422-014-0587-5

**Published:** 2014-02-06

**Authors:** Peter Gawthrop, Ian Loram, Henrik Gollee, Martin Lakie

**Affiliations:** 1 Systems Biology Laboratory, Department of Electrical and Electronic Engineering, The University of Melbourne, Melbourne, VIC 3010 Australia; 2 Institute for Biomedical Research into Human Movement and Health, Manchester Metropolitan University, John Dalton Building, Oxford Road, Manchester, M1 5GD UK; 3 School of Engineering, University of Glasgow, Glasgow, G12 8QQ UK; 4 School of Sport and Exercise Sciences, The University of Birmingham, Edgbaston, Birmingham, B15 2TT UK

**Keywords:** Intermittent control, Predictive control, Human balancing, Quiet standing

## Abstract

Two architectures of intermittent control are compared and contrasted in the context of the single inverted pendulum model often used for describing standing in humans. The architectures are similar insofar as they use periods of open-loop control punctuated by switching events when crossing a switching surface to keep the system state trajectories close to trajectories leading to equilibrium. The architectures differ in two significant ways. Firstly, in one case, the open-loop control trajectory is generated by a system-matched hold, and in the other case, the open-loop control signal is zero. Secondly, prediction is used in one case but not the other. The former difference is examined in this paper. The zero control alternative leads to periodic oscillations associated with limit cycles; whereas the system-matched control alternative gives trajectories (including homoclinic orbits) which contain the equilibrium point and do not have oscillatory behaviour. Despite this difference in behaviour, it is further shown that behaviour can appear similar when either the system is perturbed by additive noise or the system-matched trajectory generation is perturbed. The purpose of the research is to come to a common approach for understanding the theoretical properties of the two alternatives with the twin aims of choosing which provides the best explanation of current experimental data (which may not, by itself, distinguish beween the two alternatives) and suggesting future experiments to distinguish beween the two alternatives.

## Introduction

Human control strategies in the context of quiet standing have been investigated over many years by a number of authors. Early work, for example (Peterka 2002; Lakie et al. 2003; Bottaro et al. 2005; Loram et al. 2005), was based on a single inverted pendulum, single-input model of the system. More recently, it has been shown (Pinter et al. 2008; Günther et al. 2009, 2011, 2012) that a multiple segment multiple-input model is required to model unconstrained quiet standing, and this clearly has implications for the corresponding human control system. Nevertheless, the single inverted pendulum model remains of interest for two reasons: as a model of human standing where all joints except the ankle joint are physically constrained and as a simpler single-input system on which to test theories of human control. However, any such controller must also be scalable to the multiple segment multiple-input case.

Even in the ankle only model, many muscles are involved and the controlled system thus has many inputs. The theoretical and experimental elucidation of muscle synergies has also been the subject of many papers including Safavynia and Ting (2012) and Alessandro et al. (2013). Again, regarding the transformation of a single control signal to multiple muscle synergies as part of the inverted pendulum model is a useful simplification for testing theories, and this is the simplification used in this paper. But again, any such controller must be scalable to account for multiple system inputs and consequent muscle synergies.

Any real system is nonlinear, but in principle can be linearised in two stages. In the context of standing, these are determining an equilibrium joint configuration [for example using the approach of Alexandrov et al. (2005)] and then linearising the system dynamics about that equilibrium.^1^ In the single inverted pendulum case, these two steps are simple but any controller design must extend to handle the more general case. Analysis of the robustness of a controller based on such linearisation is also an issue.

Rather than address more general issues, this paper focuses on human control systems rather than the corresponding dynamics and, in particular, compares two competing control theories. To make this comparison as transparent as possible, the simple inverted pendulum model is used as a dynamical system model. However, the potential scalability of the control theories to the more general case of a multiple inverted pendulum model is a key consideration that is addressed in this paper.

A general theory of human control systems must include continuous as well as intermittent processes which incorporate discrete switching. Continuous systems integrating somatosensory, visual and vestibular sensory input are well represented by the spinal and transcortical reflexive pathways: these systems provide high-bandwidth feedback at short latency using feedback parameters which are preselected and open to modulation by multiple brain regions (Brooks 1986; Rothwell 1994; Pruszynski and Scott 2012). Switched systems selecting between multiple possibilities for movement are well represented by central selection mechanisms within the basal ganglia, prefrontal cortex and premotor cortex: these systems provide low-bandwidth feedback at longer latency using parameters selected online (Redgrave et al. 1999; Cisek and Kalaska 2005; Dux et al. 2006). Both continuous and switched systems have a primitive basis which extends through vertebrates (Redgrave et al. 1999), invertebrates (Brembs 2011) and even to the level of individual cells (Balazsi et al. 2011). In the context of human standing, continuous reflexive systems, incorporating muscle spindle and Golgi tendon organ feedback, provide tonic equilibrium joint moments through tonic stretch reflexes (Sherrington 1947) and provide partial dynamic stabilisation of the unstable mechanical system (Marsden et al. 1981; Fitzpatrick et al. 1996; Loram and Lakie 2002a, b). By itself, the continuous control system provides inadequate regulation (Marsden et al. 1981): accurate regulation requires a combined system of higher-bandwidth continuous control and lower-bandwidth control. In particular, it is suggested that intermittent control provides the lower-bandwidth central executive control driving higher-bandwidth, continuous feedback inner control loops (Karniel 2013; van de Kamp et al. 2013a). This paper focuses on the intermittent component of the combined control scheme.

Intermittent control has a long history in the physiological literature including (Craik 1947a, b; Vince 1948; Navas and Stark 1968; Neilson et al. 1988; Miall et al. 1993a; Bhushan and Shadmehr 1999; Loram and Lakie 2002a; Loram et al. 2011; Gawthrop et al. 2011). Intermittent control has also appeared in various forms in the engineering literature including (Ronco et al. 1999; Zhivoglyadov and Middleton 2003; Montestruque and Antsaklis 2003; Insperger 2006; Astrom 2008; Gawthrop and Wang 2007, 2009; Gawthrop et al. 2012).

There is a strong experimental evidence that some human control systems are intermittent (Craik 1947a; Vince 1948; Navas and Stark 1968; Bottaro et al. 2005; Loram et al. 2012; van de Kamp et al. 2013b), and it has been suggested that this intermittency arises in the central nervous system (CNS) (van de Kamp et al. 2013a). For this reason, computational models of intermittent control are important and, as discussed below, a number of versions with various characteristics have appeared in the literature.

Intermittent control action may be initiated at regular intervals determined by a clock, or at irregular intervals determined by events; an event is typically triggered by an error signal crossing a threshold. Clock-driven control is discussed by Neilson et al. (1988) and Gawthrop and Wang (2007). Event-driven control is used by Bottaro et al. (2005, 2008); Astrom (2008); Asai et al. (2009); Gawthrop and Wang (2009) and Kowalczyk et al. (2012). (Gawthrop et al. (2011), §4) discuss event-driven control but with a lower limit $\varDelta _{min}$ on the time interval between events; this gives a range of behaviours including continuous, timed and event-driven control. Thus, for example, threshold-based event-driven control becomes effectively clock driven with interval $\varDelta _{min}$ if the threshold is small compared to errors caused by relatively large disturbances. There is evidence that human control systems are, in fact, event driven (Navas and Stark 1968; Loram et al. 2012). For this reason, only event-driven control is considered in the rest of this paper.

State feedback control requires that the current system state (for example angular position and velocity of an inverted pendulum) is available for feedback. In contrast, output feedback requires a measurement of the system output (for example angular position of an inverted pendulum). The classical approach for output feedback in a state space context (Kwakernaak and Sivan 1972, Goodwin et al. 2001) is to use an observer (or the optimal version, a Kalman filter) to deduce the state from the system output. Of the biologically orientated methods considered here, that of Gawthrop et al. (2011) [based on Gawthrop and Wang (2007, 2009)] explicitly uses an observer; Bottaro et al. (2008); Asai et al. (2009) and Kowalczyk et al. (2012) do not. Because of the separation principle ((Kwakernaak and Sivan 1972, §5.3)) and ((Goodwin et al. 2001, §18.4)), this difference is not important and so, for simplicity, state feedback will be considered for the rest of this paper.

As well as introducing the concept of intermittency into the theory of physiological control, Craik (1947a) also emphasised that intermittent corrections were “ballistic” in the sense that “they have a predetermined time pattern and are ‘triggered off’ as a whole”. Ballistic control, whereby a sequence of open-loop control signal trajectories is applied to the system, is used by Neilson et al. (1988); Hanneton et al. (1997); Loram and Lakie (2002a); Montestruque and Antsaklis (2003); Bottaro et al. (2005, 2008); Astrom (2008); Gawthrop and Wang (2009) and Gawthrop et al. (2011). As the term “ballistic” has a different connotation in the area of dynamical systems, this approach will be referred to as *open-loop trajectory* (OLT), rather than ballistic, control in the sequel. In contrast, switched feedback control, where a feedback controller is switched on and off, is used by Insperger (2006); Stepan and Insperger (2006); Asai et al. (2009) and Kowalczyk et al. (2012). In the off phase, the control signal is zero. This will be referred to as *zero control* (ZC) in the sequel.

Human control systems are associated with time delays. In engineering terms, it is well known that a predictor can be used to overcome time delay (Smith 1959; Kleinman 1969; Gawthrop 1982). As discussed by many authors (Kleinman et al. 1970; Baron et al. 1970; McRuer 1980; Miall et al. 1993b; Wolpert et al. 1998; Bhushan and Shadmehr 1999; Van Der Kooij et al. 2001; Gawthrop et al. 2008, 2009, 2011; Loram et al. 2012), it is plausible that physiological control systems have built in model-based prediction. Gawthrop et al. (2011) base their intermittent controller on an underlying predictive design; Bottaro et al. (2008); Asai et al. (2009) and Kowalczyk et al. (2012) do not.

A number of computational theories of the intermittent control of quiet standing have been proposed including those of Bottaro et al. (2005, 2008); Asai et al. (2009); Gawthrop et al. (2011); Kowalczyk et al. (2012) and Suzuki et al. (2012). The papers of Bottaro et al. (2005, 2008) are precursors to the paper of Asai et al. (2009) and the paper of Suzuki et al. (2012) is a multivariable extension. The paper (Kowalczyk et al. 2012) analyses an approach closely related to (Asai et al. 2009).

The two papers (Gawthrop et al. 2011) and (Asai et al. 2009) use the term “intermittent control” in the title of the papers; this paper focuses on the similarities and differences of the theories exemplified by these two papers. Section 2 investigates differences in the control *architectures*, and Sect. 3 investigates differences in the control *behaviour* as a prerequisite for experimental testing of the two alternative hypotheses. Section 4 draws together some conclusions and makes suggestions for future work.

## Architectures

There are a number of differences between the alternative approaches discussed in the Introduction; this section focuses on one of these: OLT (open-loop trajectory control) versus ZC (zero control). For this reason, this paper uses an architecture based on that of Gawthrop et al. (2011) but with both OLT and ZC versions. The controlled system is modelled by:

1 $$\begin{aligned} \dot{x}(t)&= A x(t) + B u(t) - B_d d(t) \end{aligned}$$

where $x$ ($n \times 1$), $u$ ($n_u \times 1$) and $d$ ($n_u \times 1$) are the system state, control input and input disturbance, respectively. $A$ ($n \times n$), $B$ ($n \times n_u$) and $B_d$ ($n \times n_u$) define the system dynamics. $n$ is the system order, and $n_u$ is the number of system inputs. In the special case of the simple inverted pendulum, $n=2$ and $n_u=1$; but the method is applicable in the general case.

The intermittent control model of Gawthrop et al. (2011) is based on an *underlying continuous-time control design*. In particular, it is based on the standard linear-quadratic (LQ) control theory to be found in textbooks (Kwakernaak and Sivan 1972; Goodwin et al. 2001). LQ control has been used to model human control systems by a number of authors including Kleinman et al. (1970), Kuo (1995), Kuo (2005) and Todorov and Jordan (2002). The dual theory of optimal observers has been used for sensor integration by Van Der Kooij et al. (1999) and Kuo (2005); but, as mentioned in the Introduction, observers are not pursued further in this paper.

Human control systems contain time delays. For this reason, Kleinman (1969) extended the LQ theory to include a pure time delay ${\varDelta }$ in the controller and designed the corresponding optimal state predictor giving a prediction $\hat{x}_p(t-{\varDelta })$ of the system state $x(t)$ at time $t$ based on measurements taken up to time $t-{\varDelta }$.

The model of intermittency presented by Gawthrop et al. (2011) is based on the LQ control design extended to include time delays by Kleinman (1969). In the context of intermittent control, the predictor is particularly simple ((Gawthrop et al. 2011, §3.3)) and the prediction error $e_p$ can be written as:

2 $$\begin{aligned} e_p(t) = \hat{x}_p(t-{\varDelta }) - x(t) \end{aligned}$$

and $e_p$ is independent of $x$. The continuous-time design method underlying the intermittent control is:

3 $$\begin{aligned} u(t) = -k \hat{x}_p(t-{\varDelta }) \end{aligned}$$

where $k$ ($n_u \times n$) is the *state feedback matrix* resulting from the LQ design.

Combining Eqs. (1), (2) and (3) gives the closed-loop system:

4 $$\begin{aligned}&\dot{x}(t) = A_c x(t) - Bk e_p(t) - B_d d(t) \end{aligned}$$

5 $$\begin{aligned}&\hbox {where } A_c = A - Bk \end{aligned}$$

The LQ design method ensures that the closed-loop system matrix $A_c$ has eigenvalues with strictly negative real parts and thus corresponds to a stable system (Kwakernaak and Sivan 1972; Goodwin et al. 2001).

The *ideal* system state trajectory $x_c(t)$ is an $n \times 1$ vector function of time $t$ starting at time $t=t_i$. It is defined in terms of the closed-loop system matrix $A_c$ and the state $x(t_i)$ at the time $t_i$ as:

6 $$\begin{aligned}&\dot{x}_c(t) = A_c x_c(t) \quad \hbox { for } t > t_i \end{aligned}$$

7 $$\begin{aligned}&x_c(t_i) = x(t_i) \end{aligned}$$

In particular, the ideal system state trajectory is a trajectory leading from the current state at $t=t_i$ to the origin:

8 $$\begin{aligned}&x_c(t) = e^{A_c \tau }x(t_i)\quad \hbox { for } t \ge t_i \end{aligned}$$

9 $$\begin{aligned}&\hbox {where } \tau = t-t_i \end{aligned}$$

the $n$ components of $x_c(t)$ are thus determined, through $A_c$, by the system dynamics of Eq. (1) and the feedback gain $k$ (3) arising from the LQ design, and by the system state $x(t_i)$.

The intermittent equivalent replaces the control (3) by:

10 $$\begin{aligned}&\dot{x}_h(t) = A_c x_h(t) \quad \hbox { for } t \ne t_i \end{aligned}$$

11 $$\begin{aligned}&x_h(t_i) = \hat{x}_p(t_i-{\varDelta }) \end{aligned}$$

12 $$\begin{aligned}&u(t) = -k x_h(t) \end{aligned}$$

where $A_c$ is defined by Eq. (5) and $\hat{x}_p(t_i-{\varDelta })$ is the delayed prediction of the system state at the $i$th intermittent time point $t_i$. As discussed by Gawthrop et al. (2011), this prediction is only required at the intermittent time points, and thus, the corresponding predictor is simpler than that required for the continuous-time design of Kleinman (1969). Equation (11) means that the hold state $x_h$ is reinitialised to the continuous-time predicted state at time $t=t_i$. Equations (10) and (11) form the system-matched hold.

It is illuminating to rewrite these equations in error form by defining the *hold error* $\tilde{x}_h$ as the difference between the hold state $x_h$ and the actual state $x$ and the *state error* $\tilde{x}$ as the difference between the actual state $x$ and the ideal state $x_c$. That is

13 $$\begin{aligned}&\tilde{x}_h(t) = x_h(t)-x(t)\end{aligned}$$

14 $$\begin{aligned}&\tilde{x}(t) = x(t)-x_c(t) \end{aligned}$$

Using Eqs. (13) and (14) and rearranging Eqs. (1) and (10) gives: 2

15 $$\begin{aligned}&\dot{\tilde{X}}(t) = \left[ \begin{array}{cc} A_c &{} -Bk\\ {\mathbf {0}}_{n \times n}&{} A \end{array} \right] \tilde{x}(t) - \left[ \begin{array}{c} B_d \\ -B_d \end{array} \right] d(t) \end{aligned}$$

16 $$\begin{aligned}&\hbox {where } \tilde{X}(t) = \left[ \begin{array}{c} \tilde{x}(t)\\ \tilde{x}_h(t) \end{array} \right] \end{aligned}$$

17 $$\begin{aligned}&\hbox {and }\tilde{X}(t_i) = \left[ \begin{array}{c} {\mathbf {0}} \\ e_p(t_i) \end{array} \right] \end{aligned}$$

When $t \ne t_i$, Eq. (15) implies the open-loop trajectories of $X$ and therefore of $x$. In the ideal case that $d(t)=0$ and $e_p(t_i)=0$, the state error $\tilde{x}=\tilde{x}_h={\mathbf {0}}$ and the open- and closed-loop trajectories are the same, and the system state trajectory $x(t)$ is equal to the ideal state trajectory $x_c(t)$ of Eq. (8). In this ideal case, therefore, the state error $\tilde{x}_h={\mathbf {0}}$ and the intermittent control creates a stable manifold defined by (8) leading from the current state to the origin.

In the non-ideal case, $\tilde{x}_h\ne {\mathbf {0}}$. In this case, $\tilde{x}_h$ is generated via the *open-loop* matrix $A$, and in the case of an inverted pendulum, $A$ corresponds to an unstable system. Moreover, $\tilde{x}_h\ne {\mathbf {0}}$ drives $\tilde{x}(t)$ away from zero via the coupling term $-Bk$ in Eq. (15). For this reason, the hold error $\tilde{x}_h$ is used to generate the events $t_i$ when a new sample is taken and the error states of Eq. (15) are reset. In particular, the quadratic *switching function* is defined by

18 $$\begin{aligned} \tilde{x}_h^T(t) Q_t \tilde{x}_h= q_t^2 \end{aligned}$$

where $Q_t$ is a positive semi-definite matrix. In the special case that $x$ (and thus $\tilde{x}_h$) has only two components, two examples of $Q$ are

19 $$\begin{aligned} Q_t&= \left[ \begin{array}{c@{\quad }c} 1 &{} 0 \\ 0 &{} 1 \end{array} \right] \qquad (\hbox {Circle: } x_1^2+x_2^2=q_t^2) \end{aligned}$$

20 $$\begin{aligned} Q_t&= \left[ \begin{array}{c@{\quad }c} 0 &{} 0 \\ 0 &{} 1 \end{array} \right] \qquad (\hbox {Straight lines: } x_2 = \pm q_t) \end{aligned}$$

As mentioned in the Introduction, the ZC strategy replaces the system-matched control trajectory $u(t)$ generated by Eq. (10)–(12) by $u(t)=0$ when the system state crosses the switching surface (18). To provide a direct comparison between the two approaches, the equivalent of ZC is created within the OLT framework simply by initialising the hold state $x_h$ to zero in Eq. (11) instead of to the predicted state $\hat{x}_p(t_i-{\varDelta })$. This has the following consequences:

21 $$\begin{aligned}&\begin{array}{ll} u(t) = 0&\hbox { from } (12) \end{array} \end{aligned}$$

22 $$\begin{aligned}&\begin{array}{ll} \tilde{x}_h(t) = -x(t)&\hbox { from } (13) \end{array} \end{aligned}$$

23 $$ \begin{aligned}&\begin{array}{ll} \dot{x}(t) = A x(t) + B_d d(t)&\hbox { from } (1)\, \& \, (21) \end{array} \end{aligned}$$

Equation (21) is the required controller behaviour. Equation (22) means that the relative switching surface of Eq. (18) becomes the absolute switching surface given by

24 $$\begin{aligned} x^T(t) Q_t x(t) = q_t^2 \end{aligned}$$

Equation (23) means that the system behaviour corresponds to the (unstable) open-loop system driven by the disturbance $d$ and determined by the matrix $A$. Thus, the behaviour inside the switching surface is the same as that of the controllers of Asai et al. (2009) and Kowalczyk et al. (2012).

Outside the switching surface, Asai et al. (2009) and Kowalczyk et al. (2012) use a delayed PD (proportional $+$ derivative) controller; here, we use an intermittent controller based on state feedback. However, in the examples, the system state comprises the system angular position and velocity, and thus, state feedback (3) is equivalent to delayed PD control. As discussed by Gawthrop et al. (2011), the corresponding intermittent controller approximates the underlying *predictive* continuous controller. Thus, the essential difference between the delayed PD and the controller of this section is the use of prediction. As discussed in the Introduction, it can be argued that humans do, in fact, use predictive control.

## Behaviour

It is natural to analyse control systems incorporating switching in terms of switching surfaces and trajectories in state space. In the the case of second order systems, such analysis is represented by the well-known phase plane. Asai et al. (2009) make the crucial insight that switched control can be usefully designed to drive the system state towards stable manifolds^3^ (curves in the state space which lead to the origin) rather than towards the origin itself. Moreover, switched control of unstable systems can lead to homoclinic orbits$^{3}$ (closed curves in the state space which include an equilibrium point). As pointed out by Kowalczyk et al. (2012), these can arise when system parameters are suitably perturbed. But the notion of homoclinic orbits can also be explicitly applied to control system design in the context of unstable systems (Lozano et al. 2000). We believe that the twin concepts of stable manifolds and homoclinic orbits are key to understanding intermittent control in the context of the human standing; for this reason, the approach of Gawthrop et al. (2011) is reinterpreted in this paper in the light of these twin concepts.

Similarly, switched control of unstable systems can lead to limit cycles$^{3}$ (closed curves in the state space which do not include an equilibrium point and correspond to nonlinear oscillations); and such limit cycles are indeed predicted by Bottaro et al. (2005, 2008), Asai et al. (2009) and Kowalczyk et al. (2012). However, as explained in this paper, using open-loop trajectory (OLT), rather than zero (ZC), control leads to homoclinic orbits rather than limit cycles.

A key idea in the paper of Asai et al. (2009) is the notion of stable manifolds$^{3}$. In particular, the stable manifold is determined by the eigenvalues of the open-loop system matrix $A$ (1). As discussed in Sect. 2, the switching surface is designed to drive the system state towards this stable manifold.

This section illustrates the fact that the use of the system-matched hold based OLT control trajectories of Sect. 2 leads to system state trajectories which, in the absence of disturbances, correspond to the stable system determined by the eigenvalues of the closed-loop system matrix $A_c$ (4).

**Fig. 1:**
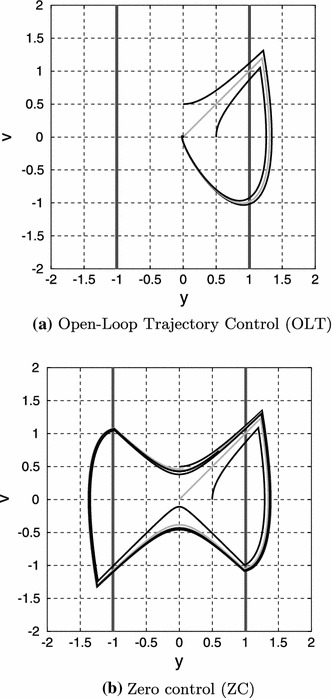
Initial condition response ($d(t)=0$). The system state trajectories starting from three initial conditions are shown. **a** All three trajectories asymptotically approach the equilibrium at the origin and the grey trajectory is part of a homoclinic orbit. **b** All three trajectories asymptotically approach a stable limit cycle

Figure 1 shows the initial condition response (with $d(t)=0$) of the two versions of the intermittent controller (OLT and ZC) using the particular parameters of Appendix 2, and the switching surface defined by Eq. (20). Figure 1a shows the two system states: velocity $x_1=v$ and position $x_2=y$; the hold state component of $X$ is not shown. Three system state trajectories are shown where the system state $x$ is initialised at 3 initial values $[0\;0.5]^T$, $[0.5\;0]^T$ (black lines) and $[0.01\;0.01]^T$ (grey line). In the case of OLT, the hold state is initialised to zero. In both cases, the unstable open-loop system drives the state onto the switching boundary. The resultant OLT signal drives the state exactly to the origin where it remains; in contrast, the ZC approach leads to each trajectory approaching a stable limit cycle. As discussed by Asai et al. (2009), the switching surface can be specially tailored to improve the performance in the ZC case.

In this particular case, and when using OLT, the initial value $[0.01\;0.01]^T$ lies on a homoclinic orbit formed from the unstable curve $x_1=x_2$ and the system state trajectory leading back to the origin. There is a similar homoclinic orbit for negative values of $x_1$ and $x_2$.

The addition of system noise will prevent the system exactly reaching equilibrium. Figure 2 corresponds to Fig. 1 except that a single system state trajectory starting at zero is plotted, and the system is perturbed by noise $d$ with standard deviation $0.1$.^4^ Although Fig. 2a *appears* to correspond to the sort of limit cycles discussed by Asai et al. (2009) and Kowalczyk et al. (2012), there are no limit cycles: Figure 2a is a perturbed version of the homoclinic orbit shown in grey in Fig. 1a. The system state trajectories using OLT and ZC are superficially similar; however, the OLT trajectory is aperiodic, whereas the ZC trajectory becomes more periodic as the variance of the disturbance $d(t)$ is reduced. This is another aspect of the *masquerading* property of intermittent control discussed by Gawthrop et al. (2011).

**Fig. 3:**
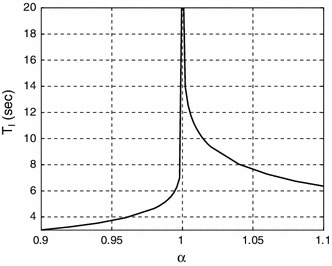
Limit cycle periods $T_l$ resulting from perturbing the open-loop trajectory controller (OLT) with parameter $\alpha$ (25). $\alpha =1$ corresponds to no perturbation, and the resultant homoclinic orbit has infinite period; small perturbations give long-period limit cycles

Similarly, the perturbation of system or controller parameters will prevent the system exactly reaching equilibrium. Figure 3 examines the case where the hold (11) matrix $A_c$ (5) is replaced by:

25 $$\begin{aligned} A_c = A - \alpha Bk \end{aligned}$$

$\alpha =1$ thus corresponds to the unperturbed case (5). In particular, the period $T$ of the limit cycle for OLT is plotted against an (unmodelled) system gain factor $\alpha$. No modelling error ($\alpha =1$) gives an homoclinic orbit; other cases give a limit cycle which, for small perturbations has a long period. Loosely speaking, the ideal case ($\alpha =1$) corresponds to an infinite-period limit cycle.

## Conclusion

Two types of intermittent control have been compared using the single inverted pendulum model of Loram et al. (2005): the ZC (zero control) approach formulated by Asai et al. (2009) and the OLT (open-loop trajectory control) approach formulated by Gawthrop et al. (2011).

The two approaches have much in common. In particular, there is an underlying continuous-time design method; there is a switching surface designed to prevent system state trajectories deviating too far from a stable manifold, and the control is open-loop when inside the switching surface.

There are three key algorithmic differences. The OLT approach uses:

1. a nonzero (though open loop) control inside the switching surface generated by the system-matched hold which, in the absence of disturbances, drives the system state towards equilibrium,
2. a switching surface based on the *relative* distance between the current state and the current stable manifold and
3. a state predictor.

The ZC approach uses:

1. a zero control inside the switching surface,
2. a switching surface based on the current state and
3. no predictor.

There are a number of ways of comparing the two approaches.

- Generality: The OLT approach has been suggested as a general model for human control systems; the ZC model just for balance. Thus, the OLT approach potentially has more explanatory power.
- Algorithmic complexity: On the one hand, the OLT approach (Gawthrop et al. 2011) is a more complex algorithm than the ZC approach (Asai et al. 2009). On the other hand, the OLT approach is simple insofar as it does not switch between two different algorithms at the switching surface; it merely chooses when to take the next sample. Moreover, the predictor is particularly simple in the intermittent case.
- Consequences: The ZC approach inevitably leads to limit cycles; the OLT approach does not (except where due to incorrect internal models) but rather gives homoclinic orbits. We conjecture that limit cycles in humans are associated with poor training and incorrect internal models, and therefore, a theory of learning is needed to fully explain behaviour; this is the subject of current research.
- Scalability: The ZC approach has been extended to the double inverted pendulum case by Suzuki et al. (2012); it is not clear how the ZC approach scales to more general situations. The OLT approach is based on a linear-quadratic (LQ) optimal control design which, as discussed in Sect. 2, is valid for arbitrary state dimension ($N>2$) and arbitrary control dimension ($n_u>1$). It is thus potentially scalable to more complex situations including multiple inverted pendulum and multiple muscle synergy models. More research is needed to develop the intermittent control approach to handle the detailed dynamical models arising from the mechanical and musculature properties of human stance.
- Experimental: As Fig. 2 indicates, it is hard to distinguish between the two approaches using only measurements of sway angle and angular velocity. However, using measurements of muscle activity, it is known zero control is *not* observed in quiet standing; in particular, it is exceptionally rare for all ankle crossing muscles to be simultaneously switched off (Di Giulio et al. 2009). This effectively rules out the ZC alternative. The challenge is to devise experiments on human standing which do lead to clear differences in the sway data. For example, it is known that double stimulus experiments distinguish between event-driven intermittent control (Loram et al. 2012), timed-intermittent control and continuous control. It is possible that a similar form or perturbation of quiet standing could distinguish between OLT and ZC. Further, as discussed by (Gawthrop et al. (2011), Section 4.2 & Appendix B), when an experiment involves smooth (that is bandlimited) disturbances, the OLT approach *masquerades* as a continuous-time controller; and this explains why the seminal experiments of Kleinman et al. (1970) could be explained by a continuous-time controller. In contrast, the ZC controller does *not* have the masquerading property and thus cannot explain the experimental results of Kleinman et al. (1970).

To summarise, we believe that the OLT approach has the theoretical advantages of generality, performance and scalablility and that this by itself is sufficient to make OLT the favoured model. Although we have shown that it would be hard to distinguish the two approaches by analysing sway data in the context of quiet standing, we suggest that additional evidence from muscle activation data does support OLT rather than ZC. We further suggest that experiments involving suitable perturbation signals could, in principle, distinguish between the two approaches.

## Appendix 1: Definition of terms

This appendix provides intuitive definitions of some mathematical terms used in this paper. For more precise definitions, see, for example, Hirsch et al. (2012).

Consider state space containing the state $X(t)$ of a dynamical system.

- Equilibrium point: $X_0$ is an equilibrium point if when $X(t_0)=X_0$, $X(t)=X_0$ for all $t>t_0$. That is, if the system is at equilibrium, it stays there.
- Stable manifold: An open curve in the state space which includes an equilibrium point $X_0$ and such that if $X(t_0)$ lies on the curve it implies that $X(t)$ stays on the curve for all $t>t_0$ and that $\displaystyle \lim _{t\rightarrow \infty }{X(t)} = X_0$. That is, if the current state lies on a stable manifold, it will move along that manifold to the equilibrium point.
- Limit cycle: A closed curve in the state space such that if $X(t_0)$ lies on the curve it implies that $X(t)$ stays on the curve for all $t>t_0$ and that there is a period $T$ such that $X(t+T)=X(t)$. In other words, on a limit cycle curve, the system state travels around that curve with a fixed period.
- Homoclinic orbit: A closed curve in the state space such that if $X(t_0)$ lies on the curve, it implies that $X(t)$ stays on the curve for all $t>t_0$ and the curve includes an unstable equilibrium $X_0$. In other words, a homoclinic orbit is like a limit cycle except that the system comes to rest at $X=X_0$ taking an infinite time to do so. It is different from a stable manifold in that it is a closed curve which starts and finishes at an equilibrium $X=X_0$.

## Appendix 2: Simulation parameters

26 $$\begin{aligned} A&= \left[ \begin{array}{ll} 0 &{} 1\\ 1 &{} 0 \end{array}\right] ,\; B = \left[ \begin{array}{l} 1 \\ 0 \end{array}\right] ,\; C = \left[ \begin{array}{ll} 0&1 \end{array} \right] \end{aligned}$$

27 $$\begin{aligned} k&= \left[ \begin{array}{ll} 2.94 &{} 4.32\\ \end{array}\right] \end{aligned}$$

28 $$\begin{aligned} {\varDelta }&= 0.18 \end{aligned}$$

In the case of Fig. 2, the system is perturbed by a multisine sequence with variance $0.1$.

## Footnotes

1 It is also possible to linearise about non-equilibrium trajectories (Hunt and Johansen 1997), although linearisations around equilibria lead to simpler physical interpretation.

2 Throughout the paper, the symbol ${\mathbf {0}}$ is the $n \times 1$ zero vector and ${\mathbf {0}}_{n \times n}$ is the $n \times n$ zero square matrix.

3 See Appendix 1.

4 The noise was generated using a random-phase multisine (Pintelon and Schoukens 2001) with a flat spectrum between 0.1 and 5 Hz.
